# The impact of COVID-19 on mentoring early-career investigators: “Everything can wait. Listen more than usual and share your own struggles”

**DOI:** 10.1097/MD.0000000000027423

**Published:** 2021-10-08

**Authors:** Mallory O. Johnson, Monica Gandhi, Jonathan D. Fuchs, Lauren Sterling, John A. Sauceda, Michael S. Saag, Elise D. Riley, Jae M. Sevelius

**Affiliations:** a Department of Medicine, University of California, San Francisco, School of Medicine, San Francisco, CA; b San Francisco Department of Public Health, San Francisco, CA; c Department of Medicine, University of Alabama at Birmingham, Birmingham, AL.

**Keywords:** AIDS, career development, COVID-19, HIV, mentoring

## Abstract

The COVID-19 pandemic disrupted almost all sectors of academic training and research, but the impact on human immunodeficiency virus (HIV) research mentoring has yet to be documented. We present the perspectives of diverse, experienced mentors in a range of HIV research disciplines on the impact of COVID-19 on mentoring the next generation of HIV researchers.

In November to December, 2020, we used an online data collection platform to cross-sectionally query previously-trained HIV mentors on the challenges related to mentoring during the pandemic, surprising/positive aspects of mentoring in that context, and recommendations for other mentors. Data were coded and analyzed following a thematic analysis approach.

Respondents (180 of 225 mentors invited [80% response]) reported challenges related to relationship building/maintenance, disruptions in mentees’ training and research progress, and mentee and mentor distress, with particular concerns regarding mentees who are parents or from underrepresented minority backgrounds. Positive/surprising aspects included logistical ease of remote mentoring, the relationship-edifying result of the shared pandemic experience, mentee resilience and gratitude, and increased enjoyment of mentoring. Recommendations included practical tips, encouragement for patience and persistence, and prioritizing supporting mentees’ and one's own mental well-being.

Findings revealed gaps in HIV mentors’ competencies, including the effective use of remote mentoring tools, how to work with mentees in times of distress, and the prioritization of mentor well-being. Mentors are in a unique position to identify and potentially address factors that may lead to mentees leaving their fields, especially parents and those from underrepresented backgrounds. We discuss implications beyond the COVID-19 pandemic.

## 1 Introduction

The importance of effective mentoring to early-stage investigators (ESIs) in academic research has been well documented.^[1–3]^ Of particular significance is the need for consistent, tailored mentoring that takes into account the challenges faced by ESIs who are attempting to build research careers. The mounting barriers to successful retention of ESIs in academic medicine, including high student loan burdens, decreased availability of tenure-track positions, and declining funding for research, have intensified this need. In early 2020, the SARS-CoV-2 pandemic introduced an unprecedented and prolonged disruption to almost every sector of the globe. In academia, the pandemic (and consequences of it even as we hopefully enter the final wave) has the potential to derail the career trajectories and mentoring of ESIs across several disciplines, particularly those within the diverse pipeline of investigators addressing critical issues in the fight against human immunodeficiency virus (HIV).^[4]^

Among COVID-19 threats are setbacks and derailments to progress made toward ending the global HIV epidemic.^[5,6]^ Moreover, the priority of the National Institutes of Health (NIH) and other funding agencies to diversify the pool of investigators from underrepresented minority backgrounds is particularly at risk, as the communities from which these investigators come are often disproportionately impacted by COVID-19 related morbidity and mortality.^[7]^ In addition, HIV researchers who formerly focused on HIV vaccines, novel therapeutics, cure strategies, social determinants of health, and behavioral prevention efforts have been pulled into important COVID-19 clinical and research endeavors. While the resulting clinical and research workforce of HIV experts pivoting their attention to COVID-19 is impressive, this redirection may result in a diminishing bandwidth to provide mentoring to ESIs focused on the still paramount challenges facing HIV prevention and treatment research.

The purpose of this paper is to explore the perspectives of experienced HIV research mentors across the United States regarding the impact of the COVID-19 pandemic on their mentoring of ESIs who are building research careers in HIV. For almost 10 years, the University of California, San Francisco Center for AIDS Research Mentoring the Mentors Program has conducted 2-day intensive mentor training workshops to systematically build mentoring competency among domestic HIV researchers with an emphasis on mentoring diverse mentees in HIV science. To date, our program has trained over 226 mentors from across the country, and the evaluation results have been described previously.^[8–11]^ By design, the Mentoring the Mentors Program concentrates on US-based HIV investigators, as the program includes a focus on NIH and other funding opportunities available primarily to US investigators. The goal of this study was to examine challenges expressed by these researchers of mentoring during the COVID-19 pandemic; positive or surprising aspects of mentoring presented by the pandemic; and recommendations made to other mentors for effective mentoring during this and future crises. To accomplish our goal, we leveraged a planned long-term evaluation of the Mentoring the Mentors Program among prior participants who had completed the training in which we collected qualitative data on experiences with mentoring during the pandemic.

## 2 Methods

### 2.1 Study design

The current investigation used a qualitative phenomenological research approach.

### 2.2 Procedures and participants

As part of the long-term, cross-sectional evaluation of mentors who participated in 1 of 7 two-day intensive HIV-related mentor training workshops between 2012 and 2020,^[8–11]^ we assessed respondents’ perceptions of the impact of COVID-19 on their mentoring practices, along with suggestions for addressing challenges associated with mentoring during the pandemic.

In November of 2020, a total of 226 unique participants who had completed at least one of our annual workshops were invited by email to complete a web-based survey via Qualtrics (Provo, UT) that included descriptive program evaluation questions, background and demographic questions, and 3 open-ended questions related to mentoring during COVID-19. Following initial invitations, we sent reminders weekly to those who had not responded and those who had started, but not completed, the survey. As part of the invitation, mentors were told that those who completed the survey would receive a US$10 gift code for an online retailer and entered into a raffle for an additional 5 US$100 gift codes, which we randomly selected and distributed following closeout of the survey.

The primary objective of the survey was to solicit data on the long-term impact of the program. However, we took the opportunity to include additional content related to mentoring during the SARS-CoV-2 pandemic by eliciting answers to the following open-ended questions:

1. What are the challenges, worries, or concerns you face while mentoring early-career investigators/trainees during COVID-19?
2. Are there any positive or surprising aspects to mentoring during COVID-19? Please explain.
3. What suggestions would you have for other mentors who are struggling with mentoring during COVID-19?

These questions were generated through discussions among the authorship team based on their experiences and concerns while mentoring during the early phases of the COVID-19 pandemic.

### 2.3 Ethics

The current investigation was conducted as part of a program evaluation, and thus ethics committee approval was not required.

### 2.4 Data analysis

Survey respondents provided open-text responses to each of these questions. These responses remained blinded to respondent identity, demographics, and other characteristics and were entered into qualitative coding and analysis software (Dedoose version 8.3.41; Los Angeles, CA) and then coded by the first and senior authors using a thematic analytic approach.^[12]^ First, the authors independently read the first 20 responses to each of the 3 questions and separately generated themes and an initial coding scheme. They then discussed and refined the preliminary codes until a set of initial codes were generated. Then they separately coded a set of 40 survey responses, which were discussed and reconciled, until revisions to the codes were finalized. All responses were then divided up for final coding. The survey also assessed demographic and background data for each respondent, which are reported as provided by respondents in order to characterize the sample. Missing data were minimal across qualitative and quantitative domains.

## 3 Results

### 3.1 Response rates

Of 226 mentors invited, 180 (80%) completed the survey. Of those who did not complete, 2 (<1%) responded that they were now retired or were no longer in a mentoring role; 32 (14%) never responded to multiple invitations; 3 (1%) opened but did not complete the survey; 9 (4%) opted out of completing the survey (an option offered through the survey platform that indicates they received the invitation but chose not to participate); and 1 (<1%) had a non-functioning email address and we were unable to locate more current contact information.

### 3.2 Respondent characteristics

**Figure 1:**
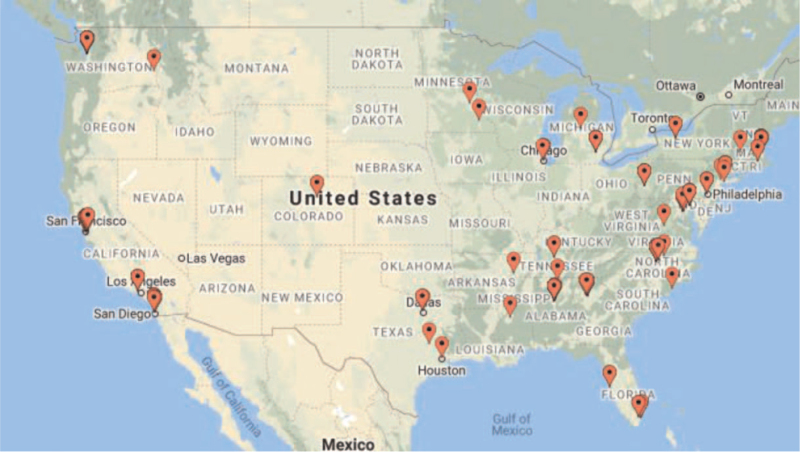
Work locations of respondents.

Respondents’ primary work locations demonstrate geographic diversity throughout the US (see Fig. 1). Table 1 presents the characteristics of the 180 mentors who completed the survey. Almost two thirds were female, 12% Black/African American, 9% Asian, and just under 8% reported Latinx ethnicity. One-fifth reported being the first in their immediate families to attend college. Forty percent reported their primary discipline as medicine, followed by public health (26%), social/behavioral science (20%), and just under 10% each as basic science and nursing. Academic ranks included full (39%), associate (39%), and assistant (17%) professors. Respondents were all faculty-level (or equivalent) investigators with a range of principal and co-investigator roles across a spectrum of HIV-related research topics spanning a range of scientific disciplines.

**Table 1:** Participant characteristics (N = 180).

		N (%)
Gender identity	Female	115 (63.2)
	Male	63 (34.6)
	Gender minority	4 (2.2)
Race^∗^	Black or African American	23 (12.4)
	Asian	17 (9.1)
	Native Hawaiian or Other Pacific Islander	0
	American Indian/Alaska Native	2 (1.1)
	White	131 (70.4)
	Another race or ethnicity	10 (5.6)
Latino/a/x or Hispanic Ethnicity		14 (7.8)
First in family to attend college		36 (20.1)
Primary discipline	Medicine	72 (40.0)
	Social or behavioral science	36 (20.0)
	Public health	47 (26.1)
	Basic science	12 (6.7)
	Nursing	9 (5.0)
	Other	4 (2.2)
Current academic rank	Assistant Professor	31 (17.0)
	Associate Professor	71 (39.0)
	Professor	71 (39.0)
	Emerita/Emeritus	3 (1.7)
	Other	6 (3.3)
In mentoring/training leadership role		115 (65.3)

Proportion of time typically spent	Mean (SD)	Range
Mentoring	13.6% (11.3)	0%–80%
Teaching	9.9% (11.2)	0%–70%
Research	55.5% (21.4)	0%–100%
Clinical	7.2% (11.4)	0%–70%
Administrative	12.7% (13.8)	0%–70%
Number of primary mentees	4.6 (4.2)	0–36
Number of secondary mentees	5.0 (3.8)	0–25

∗ For race, Ns do not add up to total, as individuals could select more than 1 option.

Approximately two thirds reported currently being in a leadership role related to mentoring or training, such as directing training programs, leading mentoring or developmental cores for research centers, or having positions in academic affairs. The mean number of current primary and secondary mentees for this group of mentors were 4.6 and 5.0, respectively. Mentors reported working with mentees from the following groups: 98% with mentees who are female; 96% with mentees from racial or ethnic minority backgrounds; 79% with mentees from socioeconomically disadvantaged backgrounds; 46% with mentees who identify as gender minorities; 83% with mentees who identify as sexual minorities; 27% with mentees with disabilities; and 74% with mentees who are the first in their immediate families to attend college.

### 3.3 Impact of COVID-19 on mentoring

The qualitative data analysis was structured around 3 domains of the survey: challenges, positive/surprising aspects, and suggestions for other mentors.

#### 3.3.1 Challenges

Respondents reported a range of challenges, concerns, and worries about mentoring during the COVID crisis. These included difficulties with establishing or maintaining inter-personal relationships with mentees, concern over mentees’ and their own well-being, awareness of disruptions in their mentees’ progress, and uncertainty about the future (see Table 2 for themes and exemplar quotes).

**Table 2:** Challenges.

Themes	Illustrative quotes	Speaker characteristics
Relationship building/maintenance	*The biggest issue is that there can’t be any accidental or incidental connection; everything has to be structured. I think because of this mentors can miss a lot.*	Male, White, MD, Prof
	*I worry about not having people ‘pop in’, so worry that some mentees are not getting their needs met.*	Male, White, MD, Prof, First
	*Another downside is not being able to take mentees for coffee or lunch during meetings or to celebrate a milestone.*	Male, Asian, PH, Assoc
Research/training interruptions	*Impact on field research activities and risk of acquiring COVID-19 as front-liner clinical researchers.*	Male, Black, MD, Assoc, First
	*Concern that our physician-scientists are being pulled more into clinical work due to pandemic and may not recover or get back on track for their research.*	Female, White, SBS, Prof
	*I worry about their success. I need them to succeed in order to succeed, and I’m generally invested in their success personally, and I just worry about funding sources, ability to complete proposed study activities, etc.*	Female, White, Nursing, Assoc
	*I think the major difficulty is it seems there is less communication between mentoring meetings than when we were all physically present. This has led to miscommunications around what work has been done and not done.*	Female, Asian, MD, Assoc
Uncertainty of future	*Concerns that graduating PhDs and advancing postdocs are facing impossible job search prospects with hiring freezes across academia and layoffs in both public and private sectors.*	Female, White, PH, Assoc
	*Current time is fairly unprecedented, so it is more difficult to project confidence in positive outcomes, for things we have little info/evidence for (how budgets such as NIH will be impacted, how job availability/security in academia will change).*	Female, White, SBS, Assoc
Mentee well-being	*A lot of the trainees (maybe all) are extremely stressed. I have a trainee crying nearly every day. I worry about having adequate support available for them.*	Female, Middle Eastern, SBS, Assoc
	*The vulnerability in terms of the mental and social well-being of early-career trainings during this time is tough to witness, and given the uncertainty of conducting future research only adds to the stress of attempting to pursue a career in research.*	Male, Latinx, SBS, Assist, First
	*The mentees are stressed out, depressed. It is hard for them to focus on their tasks.*	Male, Black, PH, Prof
	*Some mentees are experiencing a lot of stress or complications managing anxiety, depression and other disorders. Sometimes, mentorship sessions feels like therapy - and I am not trained for therapy and I do not pretend to be one - but there is a thin/complicated line between mentoring and supporting your mentee and having to help a mentee seeking professional help.*	Male, Latinx, PH, Assoc, First
	*I think there is so much uncertainty and concern right now. I am also providing more social support--creating space for people to vent and express their worries. That is challenging for me--I like to fix and move on, so I sometimes feel a bit under-prepared for the emotional things.*	Female, Black, PH, Assist
Mentor well-being	*I am severely burned out and depressed (thinking about leaving Medicine) so it makes it difficult to mentor protégées.*	Female, White, MD, Assoc
	*How to balance my own struggles with supporting mentees’ struggles – striking the right balance of vulnerability and strength.*	Female, White, PH, Assoc
	*We had tremendous stress here in NYC, all my trainees are physicians, everyone is traumatized, trying to support all these people frankly nearly killed me.*	Female, White, MD, Prof
Subgroup considerations: parents	*I am worried about the investigators who have small children at home but who still need to get work done. I can give them words of support but that is about all I can do.*	Female, White, PH, Prof
	*Early-career faculty mentees with young children face disproportionate burden (women in general facing higher childcare burden already being reflected in published literature).*	Female, White, PH, Assoc
	*Mentees with families have increased childcare responsibilities which takes away from their time and energy for their research.*	Female, White, MD, Assoc
	*The toll my mentees have at home caring for children and the mental stress they have of falling behind on their timeline.*	Female, Latinx, PH, Assist, First
Subgroup considerations: under-represented minority mentees	*Disproportionate burden on BIPOC mentees (students and faculty) because of layered challenges and strains of covid, overt acts of widespread racism and discrimination, and election-related stressors. Our students are repeatedly describing themselves as “totally exhausted”, “unable to focus”, “drained”, etc.*	Female, White, PH, Assoc
	*We are surviving a pandemic and for many people, it's been a tough year - particularly if you are Latinx, Black, or a racial/ethnic minority.*	Male, Latinx, PH, Assoc
	*Racial injustice and other politically-charged experiences bring an added layer of challenge to the mentoring environment.*	Female, White, MD, Assoc
	*Underrepresented minority researchers may be especially impacted and my fear is that we lose them to other sectors.*	Male, Latinx, SBS, Assist, First

Assist = assistant professor, Assoc = associate professor, First = first in immediate family to attend college, MD = medicine, NIH = National Institutes of Health, PH = public health, Prof = full professor, SBS = social/behavioral science.

##### 3.3.1.1 Relationship factors

Many respondents indicated that they found it challenging to connect in meaningful ways with their mentees due to the sudden forced reliance on video conferencing platforms such as Zoom. This manifested in the form of difficulty with non-verbal communication during remote mentoring and differing levels of engagement across mentees when using remote mentoring modalities. Some mentors experienced additional challenges of working with new mentees whom they had never met in person. Some expressed concerns that they may lose track of mentees during the current pandemic-related disruptions, and some lamented the loss of group dynamics among their mentees who previously had coalesced into a supportive group. Many cited the loss of informal interactions that were previously available when mentoring occurred in the same physical space, including hallway conversations, lunches or coffee with mentees, and open-door policies that are challenging to recreate remotely.

##### 3.3.1.2 Research/training disruptions

Many remarked that their mentees’ progress was adversely affected by the pandemic. Pausing of in-person research activities, effort diversion to COVID-related research or clinical activities, the inability to present and network at professional conferences, and competing demands for caregiving were cited as sources of disruptions threatening their mentees’ trajectories.

##### 3.3.1.3 Uncertainty of future

Respondents indicated that the unknown timeline of how long COVID-related disruptions would last contributed to the challenges in working with mentees during the pandemic. This also raised the potential threat to future employment opportunities for mentees and the uncertain availability of future funding for non-COVID related research topics.

##### 3.3.1.4 Well-being

Many mentors expressed concern about the psychological well-being of their mentees as well as their own. These concerns were linked to social isolation, lack of progress in former work, COVID-related fears, uncertainty of the future, and the social and political unrest that continues to accompany the pandemic.

##### 3.3.1.5 Subgroup specific considerations

Some expressed particular concern for the mental and physical health of mentees from specific groups, including those who were parents during school closures/remote learning and those from underrepresented minority groups given the heightened social and racial tensions in this country.

#### 3.3.2 Positive/surprising aspects

When asked about any positive or surprising aspects to mentoring during the pandemic, more than one-third of respondents (35%) either did not provide a response (n = 38; 21%) or explicitly stated that there were no positive elements (n = 25; 14%). However, the two-thirds who did provide responses pointed to the logistical ease of remote mentoring and the related increased capacity for mentoring across geographic distance; the deepening of relationships resulting from the shared pandemic experience; an increased enjoyment of mentoring; and heightened awareness of their mentees’ gratitude and resilience (Table 3).

**Table 3:** Positive and surprising aspects.

Themes	Illustrative quotes	Speaker characteristics
Logistical ease	*I miss the in-person interactions, but some of our mentoring groups have had fantastic turnout because no one has to worry about commuting across town to get to another meeting/building. We’ve been doing fun things too, like online jeopardy challenges and happy hours.*	Female, White, Nursing, Assoc
	*Better turn out for zoom than in-person mentoring group meetings!*	Female, White, SBS, Prof
	*I don’t know if it is a positive, but I definitely have more time in my schedule (no commute, fewer interruptions during the day) so I am able to devote more time to mentees. I also started using other methods (accountability groups, worksheets) that I always wanted to do, but didn’t think people would show up to campus for.*	Female, Black, PH, Assoc, First
Distance mentoring	*Great opportunities for mentoring from individuals at outside institutions.*	Female, White, MD, Assoc
	*Zoom mentoring has allowed me to take on, as primary mentor, a junior faculty member at an HBCU in another state (North Carolina). It has been a very effective mentoring relationship and would NOT have happened if C19 hadn’t opened both of us up to the possibilities of Zoom.*	Female, White, PH, Assist
Mixed blessings	*Convenience for all but you wonder at what costs?*	Male, Asian, MD, Assist, First
	*In many ways I am more available to mentees because we don’t have to travel to meet each other in person, but as stated above this can result in feeling additional pressure.*	GM, White, SBS, Assoc
Shared experience	*We’re all in the same boat, so I think it's easier to be sympathetic.*	Female, White, SBS, Assoc
	*Probably the most surprising aspect of mentoring during COVID-19 is that people have been much more open about their personal struggles, and have also told me that they see me as some kind of role model because I work and parent (and have a noisy dog). I think it makes them maybe feel a little better to see that there is chaos everywhere and we are all just trying to work through this time.*	Female, White, SBS, Assoc
	*Having my children at home with me for 4 months also humanized me a bit to people I think - nothing like a baby vomiting on you mid-meeting to make a mentee feel less intimated.*	Female, White, PH, Assoc
Enhanced connection	*Getting a visual glimpse into their personal lives-meeting pets, partners, seeing the art hanging in their homes.*	Female, White, MD, Assoc, First
	*What has surprised me is that video-conferencing from the comforts of our home and removing the formal structure of an office setting has created a more relaxed environment where we are both more open and communicative. I feel that seeing each other in our own environments has fostered a stronger collaboration for both of us which has translated into a very productive research relationship.*	Female, White, PH, Assist
Resilience	*It's not as nice as in person, but everyone has been very resilient in the face of the crisis and that has been uplifting.*	Male, White, SBS, Prof
	*I practice group mentoring and it has been fascinating to see how mentees share experiences, coping strategies, and a lot of compassion - with peers and me.*	Male, Latinx, PH, Assoc
Gratitude	*Mentees deeply appreciate the continued mentoring. Even when I feel awkward or less effective, I still get feedback of gratitude. I think everyone needs to feel supported these days, so I’m glad my mentees still feel supported.*	Female, White, MD, Prof, First
	*Sending follow-up emails about issues/challenges that the mentee has raised has been really positive, and seem to be much appreciated.*	Male, Asian, PH, Assoc
Enjoyment of mentoring	*It has been a positive experience to be able to provide emotional support to my mentees during this challenging time.*	GM, White, SBS, Assoc
	*I think it's been beneficial to allow everyone to take their foot off the gas a little. Many of my mentoring relationships have actually mellowed and become more supportive as I give people a little more room to take a break or take care of themselves.*	Female, White, Nursing, Assoc

Assist = assistant professor, Assoc = associate professor, First = first in immediate family to attend college, GM = gender minority, MD = medicine, PH = public health, Prof = full professor, SBS = social/behavioral science.

##### 3.3.2.1 Logistical ease

Many respondents commented on the practicality of remote mentoring technologies, which made scheduling meetings much easier than in person. Such modalities obviated the need for travel to in-person mentoring meetings, which resulted in better attendance at group and individual mentoring sessions. Similarly, the moratoria on work-related travel by many institutions made aligning of schedules much easier than before the pandemic. This flexibility was also framed as valuable in allowing some mentors to establish mentoring relationships with mentees who were geographically distant from them – relationships that may not have happened if COVID-19 had not forced them into conducting remote mentoring.

##### 3.3.2.2 Logistical ease as mixed blessing

It is important to note that the increased convenience of remote mentoring was not seen as predominantly positive. The logistical ease of arranging and attending remote mentoring sessions sometimes came with added pressure to always be available and to accept more mentees than they would otherwise. The resulting risks of burnout from long periods of video interaction (i.e., “Zoom fatigue”) and increased workloads were noted.

##### 3.3.2.3 Shared experience

Many commented on how the pandemic has imposed a dramatic shared experience between the mentor, the mentee, and others in their lives. Some described this experience as strengthening their connections with their mentees.

##### 3.3.2.4 Enhanced connections

Some noted that the shared experience of the pandemic increased their sense of connection with their mentees, as the use of video meetings from home provided glimpses into each other's personal lives that helped to humanize each other and bring them closer together in the mentoring relationship.

##### 3.3.2.5 Resilience and gratitude

Several respondents commented that the stresses of the COVID pandemic demonstrated the resilience of their mentees in times of turmoil, which heightened their respect for them and increased their optimism about their mentees’ futures. Similarly, some noted that mentees seemed highly appreciative and grateful for the mentor's efforts, even if they were sometimes just brief check-in emails, calls, or text messages.

##### 3.3.2.6 Renewed enjoyment of mentoring

Some, when commenting on the above positive aspects of the current circumstances, stated that they had an enhanced sense of enjoyment in their mentoring related to providing what they saw as much-needed support during a critical time that was highly appreciated.

#### 3.3.3 Recommendations

When asked for suggestions for other mentors who are struggling with mentoring during COVID-19, respondents offered a range of strategies (Table 4).

**Table 4:** Recommendations for other mentors.

Themes	Illustrative quotes	Speaker characteristics
Stay consistent	*Dedicate a fixed time to meet with mentees weekly.*	Female, Black, MD, Assoc
	*Set a schedule and stick to it.*	Male, White, SBS, Assistant
	*Maintain regular, pre-scheduled meetings with your mentees, and leave room in your schedule to have some time for off-topic conversations that may bring up issues that are no longer coming up due to the virtual meeting formats.*	Female, Latinx, PH, Assist, First
Increase connections and communication	*Empathize and be available - open phone call/text policy now that we aren’t physically in the same place all the time (replacing the open door policy).*	Female, Black, MD, Assoc
	*Maintain frequent communication with your mentees. It is easy to get zoom fatigue and to let your regular contact with mentees slip. To ensure wellness and to keep mentees motivated, it is important to remain engaged and to see them weekly (virtual or in-person….with social distance and mask!).*	Male, White, SBS, Assoc, First
	*Set reminders to just send “check in” emails to mentees if you are not in regular contact. Even for my mentees who are doing fine, they have always appreciated the contact.*	Male, Asian, MD, Assoc
	*A walking meeting with masks on can be an effective and liberating way to discuss “big picture issues”.*	Female, White, MD, Assoc
Use technology tools effectively	*Learn basic tips for effective video-based meetings, e.g., where to place your camera to maximize eye contact, allow for longer response latencies when you ask mentees to respond/interact, be aware of Zoom fatigue for both you and mentee and take breaks, use multiple modalities (not just you talking at them, but videos, web-based resources, etc).*	Female, White, SBS, Prof
	*Keeping an open chat platform through Microsoft Teams or similar; Time tracking apps or software works well for people; exchange of top 3 goals for the day with an accountability partner; zoom working sessions (e.g. writing sessions, virtual retreats, etc).*	Female, White, PH, Assoc
	*Set regular meetings; keep the cameras on, turn off the email, work hard to be mindful and present.*	Female, Asian, MD, Assoc
Provide support	*Stay tuned into their anxieties, reassure and try to keep them focused on what they can do rather than spinning out of control with all the things that they can no longer do.*	Female, Black, MD, Assoc
	*I like building in shared experiences of stressors and positive events to highlight what people are doing to manage the stress of COVID-19 and that they are not alone (often times) in their experiences of feeling isolated, becoming zoom fatigued, etc.*	Male, Latinx, SBS, Assist, First
	*No matter how difficult or uncomfortable you may feel, provide space for your mentees to express their fears and vulnerabilities.*	Female, Black, PH, Assist
	*Let others know it's challenging and uncertain and okay to feel and share that. Hold on to hope for a more settled future, but be open to new opportunities in the current moment which are demanding attention for all the right reasons. Still, such sudden changes in professional focus require a bit of grieving, even if this only turns out to be temporary. IT is a temporary loss or death even of expectations for work we were deeply invested in, but now must put off (all of us to varying degrees) to meet the current existential crisis.*	Male, White, MD, Prof, First
Slow down	*Remember to remind folks that this is a difficult time and to give themselves a break.*	Female, White, MD, Prof
	*Having reasonable expectations of trainees [and] recognize dual pandemic (blatant hate/racism policing and covid) impacts BIPOC differently.*	Female, Black, PH, Assoc, First
	*Be patient. We are surviving a pandemic and for many people, it's been a tough year -particularly if you are Latinx, Black, or a racial/ethnic minority. Everything can wait. Listen more than usual and share your own struggles. Mentees need to know they are not alone and that people they admire and respect are also struggling.*	Male, Latinx, PH, Assoc
	*I think it's been beneficial to allow everyone to take their foot off the gas a little. Many of my mentoring relationships have actually mellowed and become more supportive as I give people a little more room to take a break or take care of themselves.*	Female, White, Nursing, Assoc
	*Recognize that the burden of non-work related responsibilities has quadrupled for some, including many women who care for children.* *Recognize disparities on the effects of COVID on your mentees.*	Female, White, MD, Prof
Be persistent	*Just keep doing it. Even if you feel like you’re not as effective, your efforts really are appreciated and needed, so keep going.*	Female, White, MD, Prof, First
	*Keep up the calm and positive attitude to empower your mentees, we have to stay strong to support them.*	Female, White, MD, Prof
Self-care	*Take a few moments to breathe and meditate. Self-care is important and will ensure that you are able to provide the best COVID-19 era version of oneself during mentoring meetings with mentees.*	Female, Black, PH, Assoc, First
Get mentoring support	*Talk to other mentors.*	Female, Latinx, MD, Prof
	*I think we should be gathering and sharing wisdom and tips. And yet no one I know has more time for gathering and sharing wisdom. ARGH.*	Female, White, MD, Assoc

Assist = assistant professor, Assoc = associate professor, First = first in immediate family to attend college, MD = medicine, PH = public health, Prof = full professor, SBS = social/behavioral science.

##### 3.3.3.1 Consistency

Several respondents suggested that consistency and persistence were critical to effective mentoring during the pandemic. This included establishing and adhering to weekly schedules for each mentee, even if there are no pressing deadlines and even if it appeared that progress had been minimal.

##### 3.3.3.2 Increase connections and communications

In addition to the need for consistent, regular mentoring meetings, many mentors suggested additional and frequent check-ins between meetings to take the place of hallway conversations or other impromptu interactions that would be occurring if mentors and mentees were working on site together. Some suggested drop-in Zoom office hours or being available at specific times by phone or text if questions arose between formal mentoring meetings. Some suggested walking, outdoor, masked, in-person meetings to augment the less intimate Zoom meetings.

##### 3.3.3.3 Use technology tools effectively

Some respondents recommended that others learn how to use Zoom and other remote tools more effectively, including camera placement, use of screen sharing, annotation, and open chat features on team software applications, and recognizing the need for more breaks during remote compared to in-person meetings.

##### 3.3.3.4 Provide support

A common theme across respondents was the importance of taking the time to check in with mentees on how they were doing mentally and physically. This took the form of brief check-ins at the beginning of each mentoring session, acknowledging and normalizing the unprecedented stress of current circumstances, and providing space for mentees to see the mentor's own struggles with the pandemic.

##### 3.3.3.5 Slow down

Several respondents indicated the importance of slowing down and allowing more time for tasks than prior to the pandemic and of validating the forces that have caused delays in mentees’ progress. This was especially true for mentees who were struggling psychologically and those who had increased childcare responsibilities.

##### 3.3.3.6 Be persistent

While recognizing the need to slow down, several mentors noted that slowing the pace should not be confused with pausing or giving up on mentees. Rather, several stated the importance of persistence and “hanging in there,” and remaining a stable and supportive presence in mentees’ lives.

##### 3.3.3.7 Self-care

Consistent with the prior theme of mentor well-being, several respondents noted the importance of mentor self-care during times of stress, with the implication that if mentors did not take care of themselves, they would not be an effective resource nor source of support for mentees.

##### 3.3.3.8 Get mentoring support

Finally, some suggested that mentors reach out to other mentors to share strategies for mentoring during stressful times. As 1 respondent said: *“I think we should be gathering and sharing wisdom and tips. And yet no one I know has more time for gathering and sharing wisdom.”*

## 4 Discussion

This survey reports qualitative findings from 180 mentors across the U.S. who had previously attended a designated Mentoring the Mentors training workshop at University of California, San Francisco for HIV researchers, and showed how the pandemic has affected mentoring, offering both challenges and solutions. Among the key findings are the need for additional supports and tools for mentors working in times of disruption. Academic programs should help mentors stay consistent with mentoring, even remotely; provide support and offer stories of personal challenges during the pandemic; build mentor capacity to use remote tools more effectively; and validate productivity delays while maintaining persistence. Like most other aspects of life, the COVID-19 pandemic has disrupted mentoring of ESIs working to build careers in academic research, making the need for effective mentoring all the more important.

Although the current survey focused on the specific impact of the COVID-19 pandemic on mentoring, the pandemic occurred alongside other broad societal challenges related to racial injustice and political divisiveness. Indeed, 2020 saw a convergence of pandemic-related academic disruption, social unrest, financial uncertainty, and political turmoil that created a context unlike any other in history. Understanding the collective impact of these forces on mentoring is paramount. While there have been published perspective essays and opinion pieces on the topic,^[13–17]^ this report is the first to provide data from a large number of geographically diverse and experienced mentors on the impact of COVID-19 on their mentoring practices. It also demonstrates the position from which mentors may witness and intervene on the processes by which the COVID pandemic may differentially impact the careers of women and other caregivers,^[18]^ as well as scientists from underrepresented backgrounds.

In terms of challenges, first, with the exception of a few respondents who had previously been doing considerable virtual mentoring, moving to full-time remote mentoring felt abrupt and difficult. This illuminated a major gap in mentors’ competencies related to the effective use of remote technologies for mentoring. These competencies relate to technical challenges and to relational challenges for building and maintaining effective and productive mentoring relationships on virtual platforms. Second, the findings from this analysis revealed a notable gap in mentors’ perceived capacity to identify and manage mentee distress during turbulent times. This may be due to discomfort with the topic area, difficulty connecting via remote methods, or most likely both. Third, the confluence in research delays, sudden diversions of effort to childcare or clinical work, and anxiety about the future created an environment in which mentees were not meeting targets, and mentors did not always know how to help. Finally, the data showed that mentors were not always able to prioritize their own self-care during stressful times, a dynamic that can lead to burnout and other negative consequences. The mentors surveyed offered a range of suggestions for addressing these gaps, and the findings provide a rationale for ongoing, targeted mentoring training in these areas.

In terms of solutions, a number were offered including consistency with mentorship, persistence while acknowledging reductions in productivity, support, sharing personal stories of hardship during the pandemic, and becoming more versed with virtual tools. We propose that designated training for mentors to react nimbly to barriers to mentee progress, including the use of adaptive Individual Development Plans, may be warranted to better equip mentors in the future. Likewise, the pandemic gave mentors a look inside the home lives of many of their mentees, which allowed a vantage point into family and personal life contexts. Finding ways to better appreciate the non-work challenges that mentees face is non-pandemic times may be beneficial in providing the support and structure that is needed. Findings also support the importance of mentors being aware and connected to local, institutional, and other resources for mentees struggling during such challenging periods. These may include mental health resources, childcare options, and financial support to offset delays in training and research offered by the institution, locality, and professional organizations.

There are limitations that should be considered when generalizing the results. The mentors surveyed in this study had self-selected to both previously participate in an intensive in-person mentor training workshop and also agreed to complete the survey when invited. Therefore, the current sample may represent mentors that are particularly dedicated to mentoring ESIs in HIV research. Likewise, those who did not respond may have been particularly affected by the COVID-19 pandemic to the extent that they did not have the time or bandwidth to participate. The online survey format allowed for the efficient text entries used in the present analyses. However, the relatively brief responses may not have fully captured the rich and nuanced underlying themes that might have been better expressed through in-depth interviews with structured probing of relevant content. The pool of mentors surveyed reflected investigators who, at least at the time of their participation in the Mentoring the Mentors Program, were based at US institutions. Therefore, the perspectives of non-US-based mentors were not evaluated. Likewise, by design, the data represent a narrow slice in time during the COVID-19 crisis, which allows for a specific vantage point, but does not reflect the challenges yet to be faced as the pandemic enters subsequent stages, such as the roll-out of vaccines and return to in-person or hybrid mentoring practices. We also did not directly query about specific subgroups of mentees, such as women, parents, and sexual and gender minorities; such a focus may have revealed more nuanced data pertinent to those groups. Finally, although this paper focuses on the perspectives of mentors working primarily in HIV research, the findings ostensibly are not confined to this context. Rather, the results are more general to the challenges of mentoring across a wider range of academic research mentoring settings. In spite of these constraints, we contend that the findings presented from this analysis offer a unique and valuable contribution to the mentoring literature and illuminate strategies and opportunities to offer effective mentoring during times of crisis.

In conclusion, findings reported here reveal a range of factors that affected HIV research mentoring resulting from an unprecedented pandemic leading to global disruption. The forced catalysis of remote mentoring, the shared experience of the pandemic, and the simultaneous threats to resources and well-being have clear impacts on mentoring in a way that provides a natural laboratory to examine what works and what does not in HIV research mentoring. While such a set of circumstances is unique and time limited, the perspectives of experienced mentors in this context have the potential to inform future efforts to build mentoring capacity even in the absence of such extraordinary events.

## Author contributions

MJ conceived of the study and led the data collection, analysis, and writing. MG, JF, JAS, ER, LS, and MS collaborated on the collection of data and drafting of the manuscript. JMS collaborated on the data analysis and interpretation and drafting of the manuscript.

**Conceptualization:** Mallory O. Johnson, Monica Gandhi, Jonathan D. Fuchs, John A. Sauceda, Michael S. Saag, Elise D. Riley, Jae M. Sevelius.

**Data curation:** Mallory O. Johnson, Jonathan D. Fuchs, Jae M. Sevelius.

**Formal analysis:** Mallory O. Johnson, Jae M. Sevelius.

**Funding acquisition:** Mallory O. Johnson, Monica Gandhi, Michael S. Saag.

**Investigation:** Mallory O. Johnson, Lauren Sterling, Elise D. Riley.

**Methodology:** Mallory O. Johnson, Lauren Sterling, John A. Sauceda, Jae M. Sevelius.

**Project administration:** Mallory O. Johnson, Lauren Sterling.

**Resources:** Mallory O. Johnson, Monica Gandhi, Lauren Sterling, Michael S. Saag.

**Software:** Jae M. Sevelius.

**Writing – original draft:** Mallory O. Johnson, Jae M. Sevelius.

**Writing – review & editing:** Mallory O. Johnson, Monica Gandhi, Jonathan D. Fuchs, Lauren Sterling, John A. Sauceda, Michael S. Saag, Elise D. Riley, Jae M. Sevelius.
